# Right Atrial Contraction Strain Is Associated With Clinically Significant Cellular Rejection in Patients After Heart Transplantation

**DOI:** 10.3389/ti.2025.14174

**Published:** 2025-10-16

**Authors:** Andreas J. Rieth, Isabella Fest, Katharina Classen, Yeong-Hoon Choi, Steffen D. Kriechbaum, Till Keller, Samuel T. Sossalla, Christian W. Hamm, Ulrich Fischer-Rasokat

**Affiliations:** ^1^ Department of Cardiology, Kerckhoff-Klinik, Justus Liebig University Giessen, Bad Nauheim, Germany; ^2^ German Center for Cardiovascular Research (DZHK), Partner Site Rhine-Main, Frankfurt am Main, Germany; ^3^ Department of Cardiac Surgery, Kerckhoff-Klinik, Bad Nauheim, Germany; ^4^ Department of Cardiology, Justus Liebig University Giessen, Universities of Giessen and Marburg, Giessen, Germany

**Keywords:** heart transplantation, cellular rejection monitoring, endomyocardial biopsy, strain echocardiography, right atrial contraction strain

## Abstract

Strain echocardiography (SE) may be used for surveillance in patients after heart transplantation (HTx); however, data on atrial strain are lacking. We aimed to compare the significance of ventricular and atrial strain with respect to an associated acute cellular rejection (ACR). Patients who underwent an endomyocardial biopsy (EMB) within 1 year after HTx were eligible for this retrospective analysis. The relationship between SE and ACR was assessed. EMB results of 52 patients (median age, 53 years; 63% male) at a median of 181 days post-HTx were identified. Mild ACR was present in 19 patients and ≥ moderate ACR in 6 patients. ACR ≥ moderate was associated with right ventricular free wall strain (OR 1.20, 95%CI 1.02–1.46, P = 0.04) and right atrial contraction strain (RASct; OR 1.55, 95%CI 1.18–2.43, P = 0.01). The RASct cut-off value of −9.3% had a sensitivity of 100% and a specificity of 79% for ≥ moderate ACR. None of these associations were observed for left ventricular or left atrial strain. A validation analysis was performed on another group of 23 HTx patients, which yielded similar results with regard to the specified RASct cut-off value. Our comprehensive strain analysis confirmed the association between reduced right ventricular strain and ACR and further identified robust associations between RASct and ACR. Right atrial strain analysis may be a promising method for excluding subclinical ACR after HTx.

## Graphical Abstract

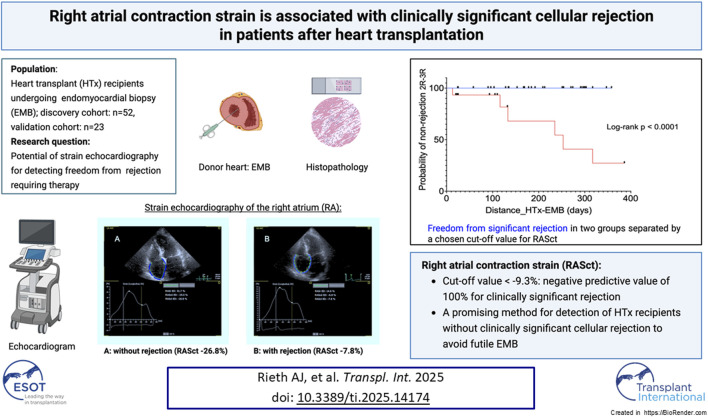

## Introduction

Rejection surveillance after heart transplantation (HTx) performed by routine endomyocardial biopsy (EMB) without clinical signs is currently a matter of debate, as the detection rates of moderate or higher-grade acute cellular rejection (ACR) requiring therapy seem low (1%–2%) in asymptomatic patients receiving standard immunosuppressive therapy [1–4]. However, rejection can occur in asymptomatic patients, particularly in the early post-transplant phase, with possible negative effects on the outcome; ACR was among the most important direct contributors to mortality within 1 year after HTx in a registry [4–6]. Therefore, there is an intensive search for non-invasive alternatives to EMB, which is performed periodically by many centers in the first 3–12 months post HTx in accordance with current guidelines [1, 4] but can have significant complications.

Among various approaches as alternatives to EMB, such as blood biomarkers, gene expression profiling, and donor-derived cell-free DNA, imaging modalities including cardiac magnetic resonance imaging (MRI) and echocardiography have been proposed [1, 7–10]. However, although several positive findings concerning strain echocardiography (SE) of the left and right ventricle and rejection monitoring after HTx exist [6, 11–16], there are no corresponding guideline recommendations for adults without significant restrictions [1]. To date, only one report on atrial SE and rejection has been published, which pertains to the left atrium [17].

The aim of the present study was to determine the usefulness of comprehensive SE analysis of both the atria and ventricles for rejection monitoring after HTx using a novel strain analysis software.

## Materials and Methods

### Patient Population

We performed a monocentric, retrospective analysis of patients undergoing EMB between 2010 und 2024 at our Heart and Thorax Center for routine surveillance after HTx. All patients underwent bicaval anastomosis at the HTx and had sinus rhythm at the time of echocardiography. Key inclusion criteria were EMB within 12 months after HTx and echocardiographic examination of sufficient quality for strain analysis within a maximum of 3 weeks before or after EMB. Patient records were screened for EMB results and the corresponding echocardiograms (Supplementary Figure S1). The study conformed to the principles outlined in the Declaration of Helsinki. All the enrolled patients signed an informed consent form for this study. Data collection and analyses were approved by the responsible Ethics Committee (protocol no. 54/12; June 5th, 2012).

### Echocardiography

All patients underwent transthoracic echocardiography in the left decubital position using a Philips, iE33 ultrasound system (Koninklijke Philips N.V., Amsterdam, Netherlands). In the majority of patients, echocardiography was performed on the day of EMB. Echocardiographic parameters were analyzed offline using dedicated software (AutoSTRAIN, TOMTEC-ARENA TTA2 2022, TOMTEC^®^ imaging systems GmbH, Unterschleissheim, Germany; distributed by PHILIPS Ultrasound Workspace) according to international standards [18] and the manufacturer’s specifications (Ultrasound Workspace (TTA2.50) AutoStrain Quick Guide December 2021).

A minimum of two recorded cardiac cycles of each view were required; however, the analyses were ultimately carried out on the cycle with the best quality. The following parameters were analyzed in accordance with current recommendations [19–21]: left ventricular (LV) systolic and diastolic volumes and volume-derived ejection fraction (LVEF), tricuspid annular plane systolic excursion (TAPSE), LV global longitudinal strain (endocardial, averaged from: apical four-chamber, apical two-chamber, and apical three-chamber view), endocardial right ventricular longitudinal strain of the free wall (RVFWSL) and of the “global 4-chamber contour” i.e., including the septum (RV4CSL), endocardial left (LA) and right atrial (RA) reservoir strain (Sr), conduit strain (Scd), and contraction strain (Sct).

**FIGURE 1:**
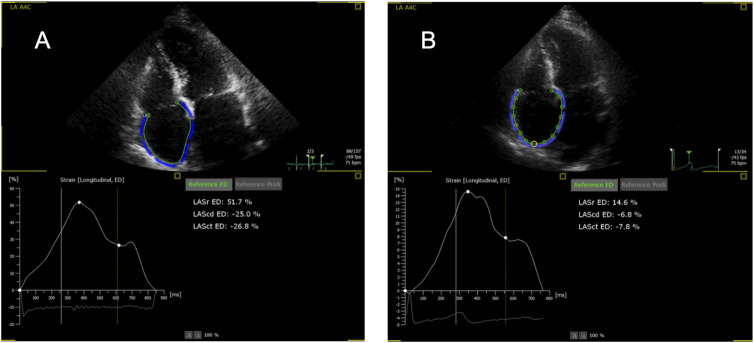
Representative examples of right atrial strain imaging in one patient. **(A)** Without rejection (0R); **(B)** With rejection (2R). Note the wrong labeling as “LA” (left atrium), for the LA program is used for the right atrium. Sr, reservoir strain; Scd, conduit strain; Sct, contraction strain.

Initially, the adequacy of the automatic tracking of cardiac cycles was reviewed and corrected, if necessary. The endocardial borders of the ventricles and atria were outlined at end-diastole and end-systole in two alternative ways. The first was fully automatic (FA), without any correction by the examiner. If the FA measurement was roughly outside the anatomical boundaries, it was classified as insufficient and not used for analysis. Second, in a semi-automatic (SA) mode, with manual setting of two markers at the left and right base of the respective cardiac chamber and then automatic outlining of endocardial borders without any correction. RA measurements were conducted only via the SA mode, because the FA mode is only available for the LA; the use of LA strain software in the SA mode for measurements of RA strain is formally off-label but available by the manufacturer and used by other groups [22]. The R wave was used as a reference, and thus zero strain was set at the R wave as recommended [18]. Examples of RA measurements are shown in Figure 1.

Three different quality levels were defined as follows: insufficient (level 0), moderate (level 1) and good (level 2) quality echocardiographic recordings. The SA measurements were repeated at least three times to achieve three results with a deviation of <10%, and the median value of these three was used for analysis. Quality level 2 was assigned when a maximum of four measurements were sufficient to achieve this goal. If more than four measurements were necessary, Level 1 was chosen. Level 0 and thus exclusion from analysis, was issued if deviation <10% was not achieved by a maximum of nine measurements or in the FA mode, as outlined above. Because of the complexity of this procedure to achieve reproducible results as far as possible, analyses to determine interobserver variability were not carried out.

### Outcomes

The revised criteria for ACR as defined by the International Society for Heart and Lung Transplantation (ISHLT) in 2004 were used as the primary outcome [23]. We analyzed the relationship between echocardiography (conventional parameters as described above and SE) and a) serial ACR grading (0R = no rejection, 1R = mild rejection, 2R = moderate rejection, 3R = severe rejection) as well as b) classification of patients as rejection requiring therapy (2R or 3R) versus patients with no need for therapy (0R or 1R) [1]. Histological examination of EMB specimens was carried out by an expert pathologist blinded to echocardiographic findings.

### Statistical Analysis

Data are expressed as mean ± standard deviation or median [interquartile range] for normally or non-normally distributed parameters, respectively. Adherence to a Gaussian distribution was determined using the Shapiro-Wilk test. Statistical significance was set at P < 0.05. For independent samples, comparisons were made using the Mann-Whitney U test for non-normally distributed parameters, the Student’s t-test for normally distributed parameters, and the Pearson Chi-squared test or Fisher’s exact test for categorical parameters.

Two separate analyses were performed. Analysis 1 included associations between echocardiographic parameters and a single EMB result within 12 months after HTx (any rejection grade and 0R/1R versus 2R/3R) that were assessed using simple logistic regression and calculation of odds ratios (OR). Receiver operating characteristics (ROC) analysis with the calculated area under the curve (AUC) was used to describe the association of a variable with endpoints. Group-wise comparisons of all rejection grades and RV4CSL were performed using ANOVA. Additionally, the probability of rejection 2R/3R in was assessed in two groups of patients divided according to an RA contraction strain (RASct) cut-off value with the highest sensitivity for 2R/3R using the Kaplan-Meier method.

Subordinate Analysis 2 pursued a different concept that focused on intraindividual SE variations depending on the rejection status. Patients for this substudy were selected if serial pairs of EMB and corresponding echocardiograms were available at different time points, preferably patients with intraindividual different EMB results (one EMB without rejection (0R) and one with rejection (1R, 2R, or 3R)). Here, the time interval between the HTx and EMB was not considered. Patients without any rejections and those without at least one 0R EMB were excluded from Analysis 2. For these analyses, the paired t-test or Wilcoxon test was used. Additionally, six individuals were analyzed, in whom one 2R or 3R EMB was available, as well as one 0R or 1R EMB. In a further modification (“extended cohort” with one additional patient previously not analyzed because no EMB within 12 months after HTx was available), we searched for every EMB with the result 2R or 3R in the patients of our study cohort, including several different EMB of the same patient, if present. The echocardiograms corresponding to those EMB were then compared with all echocardiograms corresponding to the 0R or 1R EMB in Analysis 2. For these analyses, we used an unpaired t-test or the Mann–Whitney U test.

Finally, RASct was assessed in a further cohort of patients (validation cohort) using a chosen cutoff value with regard to absence of rejection requiring therapy (Analysis 3). Statistical analyses were performed using GraphPad Prism version 10.0.2 (171) 2023.

## Results

### Analysis 1

#### Clinical Characteristics and Echocardiographic Findings

**FIGURE 2:**
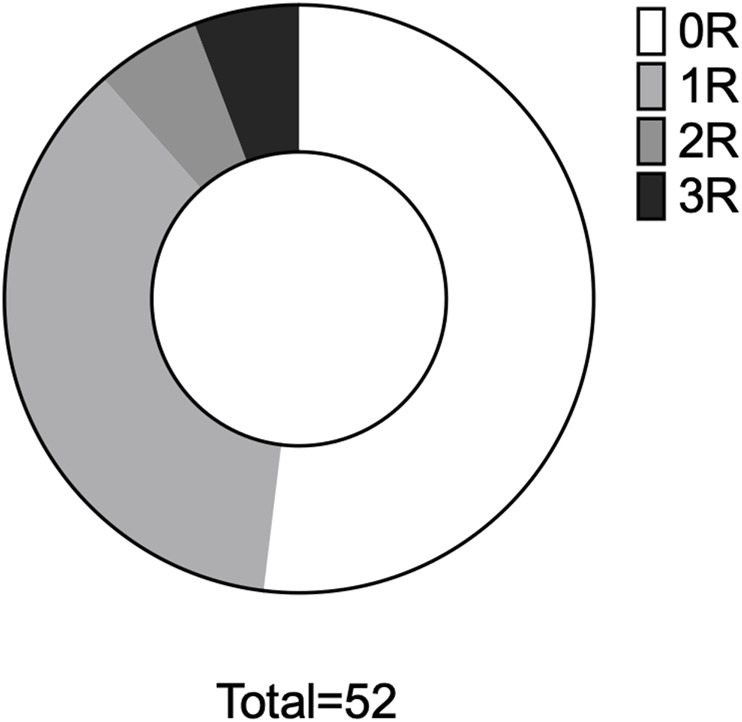
Analysis 1 - rejection proportions. Proportional distribution of rejection in the entire cohort. 0R, rejection ISHLT 0R, etc.

Of 62 patients initially screened, 52 were included in the final analysis (Figure 1): 63% were male, with a median age of 53 [47–62] years (Table 1). All the patients were asymptomatic and underwent routine EMB without a clinical trigger. All patients were in sinus rhythm. The median period between HTx and EMB was 181 [104–298] days; 27 (52%) were completely free from any rejection, 19 (36.5%) had rejection 1R, and rejection 2R or 3R was present in six individuals (11.5%) (Figure 2), all of whom were male. Standard immunosuppression with tacrolimus and mycophenolate was used in 92% of the patients, and 81% were on steroids. Echocardiography was performed mostly on the same day as EMB (81%), the quality of scans was good [2] in 87% of the recordings. Mean LVEF was 60% (±6.0), and mean TAPSE was 15.0 mm (±3.2). Significant differences between patients with rejection 0R or 1R versus 2R or 3R were found for three of the four RV strain parameters and for RASct (Table 2).

**TABLE 1:** Baseline characteristics of all patients.

Variable	All (n = 52)	No significant rejection (R0-1^a^) (n = 46)	Rejection (R2-3^a^) (n = 6)	P value^b^
Time period HTx to biopsy, days	181 [104,298]	181 [101–300]	184 [90–269]	0.7^c^
Male/female, n/n (%)	33/19 (63/37)	27/19 (59/41)	6/0 (100/0)	0.07^d^
Age, years	53 [47–62]	53 [48–62]	52 [32–59]	0.6^c^
Height, m	1.7 ± 0.11	1.7 ± 0.11	1.8 ± 0.07	0.4^e^
Weight, kg	71.0 ± 13	70.0 ± 13	79.0 ± 15	0.1^e^
BMI, kg/m^2^	23.0 ± 3.8	23.0 ± 3.8	25.0 ± 4.5	0.2^e^
HTx diagnosis				0.08^d^
DCM, n (%)	33 (63)	31 (67)	2 (33)	
ICM, n (%)	16 (31)	12 (26)	4 (67)	
Others, n (%)	3 (6)	3 (7)	0 (0)	
Clinical characteristics				
Hypertension, n (%)	33 (63)	28 (61)	5 (83)	0.4^d^
Diabetes mellitus, n (%)	10 (19)	8 (17)	2 (33)	0.3^d^
COPD, n (%)	2 (3.8)	2 (4)	0 (0)	1.0^d^
CAV >12 months after HTx, n (%)	6 (11.5)	4 (9)	2 (33)	0.1^d^
Pacemaker, n (%)	5 (9.6)	3 (7)	2 (33)	0.1^d^
Immunosuppressive treatment^f^				
TAC/MMF, n (%)	48 (92)	43 (93)	5 (83)	0.4^d^
Prednisone, n (%)	42 (81)	36 (78)	6 (100)	0.6^d^
Prednisone dose, mg	5.0 [5.0–5.6]	5.0 [5.0–17.0]	5.0 [5.0–5.0]	0.3^c^
Outcomes				
Any rejection, n (%)	25 (48)			
Rejection grade 2–3R, n (%)	6 (11.5)			
Death, n (%)	8 (15)	7 (15)	1 (17)	1.0^d^
Follow-up time, years	7.5 ± 2.4	7.4 ± 2.3	8.5 ± 3.1	0.3^e^

Values represent n (%), mean ± standard deviation, or median [interquartile range].

^a^ Refers to the 2004 ISHLT revised classification.

^b^ Rejection (R2-3) vs. no significant rejection (R0-1).

^d^ Fisher´s exact test.

^c^ Mann-Whitney test.

^e^ Unpaired t-test.

^f^ At the time of biopsy.

Abbreviations: BMI, body mass index; HTx, heart transplantation; DCM, dilated cardiomyopathy; ICM, ischemic cardiomyopathy; COPD, chronic obstructive pulmonary disease; CAV, cardiac allograft vasculopathy; AF, atrial fibrillation; TAC/MMF, tacrolimus and mycophenolate mofetil or mycophenolic acid.

**TABLE 2:** Hemodynamic and echocardiographic analyses.

Variable	n	All	No significant rejection (0R-1R^a^) (n = 46)	Rejection (2R-3R^a^) (n = 6^b^)	P value^c^
Time difference biopsy-echo, days	52	0.0 [0.0–0.0]	0.0 [0.0–0.0]	3.0 [0.0–7.5]	0.007^d^
Maximum difference, days		15.0	14.0	15.0	
Heart rate,/min	52	82 ± 11	81 ± 10	85 ± 19	0.4
CO, L/min	28	4.82 [4.1–5.79]	4.80 [4.13–5.78]	5.27 [3.70–7.87]	0.8
SPAP, mmHg	28	30 ± 8	30 ± 7	27 ± 11	0.5
PAWP, mmHg	28	11 ± 6	11 ± 6	10 ± 8	0.7
RAP, mmHg	28	6 [2–9]	5	6	0.5
PVR, WU	28	1.84 ± 0.97	1.88 ± 1.02	1.53 ± 0.18	0.6
LV Analyses					
LVEF biplane, %	50	60.0 ± 6.0	61.0 ± 5.7	56.0 ± 6.9	0.07^e^
LVEDV biplane, ml	50	103.0 [87.0–118.0]	102.0 [86.0–117.0]	111.0 [88.0–127.0]	0.6^d^
LVESV biplane, ml	50	39.0 [34.0–53.0]	38.0 [34.0–48.0]	48.0 [36.0–62.0]	0.2^d^
LVSV biplane, ml	50	63.0 [51.0–73.0]	63.0 [51.0–73.0]	58.0 [52.0–67.0]	0.6^d^
LV Strain (GLS)					
SA-peak avg	50	−17.0 ± 3.7	−17.0 ± 3.5	−15.0 ± 4.6	0.3^e^
FA-peak avg	50	−14.0 [−17.0 to −11.0]	−14.0 [−17.0 to −11.0]	−12.0 [−15.0 to −9.0]	0.2^d^
RV Analyses					
TAPSE, mm	52	15.0 ± 3.2	15.0 ± 3.0	17.0 ± 4.4	0.3^e^
RV Strain					
SA-RVFWSL	52	−20.0 ± 5.2	−21.0 ± 5.0	−16.0 ± 4.7	0.03^e^
SA-RV4CSL	52	−17.0 ± 4.1	−18.0 ± 3.8	−14.0 ± 4.5	0.03^e^
FA-RVFWSL	51	−16.0 ± 5.8	−16.0 ± 5.6	−12.0 ± 6.7	0.08^e^
FA-RV4CSL	51	−14.0 ± 4.1	−14.0 ± 3.9	−10.0 ± 4.9	0.03^e^
LA Analyses					
SA-LASr	50	25.0 [18.0–35.0]	25.0 [18.0–36.0]	18.0 [14.0–31.0]	0.3^d^
SA-LAScd	50	−14.0 [−20.0 to −10.0]	−14.0 [−20.0 to −10.0]	−14.0 [−19.0 to −11.0]	0.97^d^
SA-LASct	50	−9.4 [−14.0 to −5.2]	−9.5 [−14.0 to −5.5]	−4.4 [−12.0 to −3.1]	0.1^d^
FA-LASr	49	22.0 ± 9.7	23.0 ± 10.0	20.0 ± 8.3	0.5^e^
FA-LAScd	49	−14.0 ± 6.7	−14.0 ± 6.9	−14.0 ± 5.6	0.99^e^
FA-LASct	49	−7.0 [−13.0 to −4.9]	−7.6 [−13.0 to −5.0]	−6.3 [−10.0 to −2.6]	0.3^d^
RA Analyses					
RA area, cm^2^	49	16.3 ± 4.5	16.2 ± 4.7	17.8 ± 2.1	0.4
SA-RASr	49	31.0 ± 11.0	32.0 ± 10.0	23.0 ± 14.0	0.06^e^
SA-RAScd	49	−16.0 ± 8.4	−16.0 ± 7.9	−17.0 ± 12.0	0.8^e^
SA-RASct	49	−15.0 ± 7.6	−16.0 ± 7.2	−5.7 ± 2.4	0.002^e^

Values represent mean ± standard deviation or median [interquartile range].

Except TAPSE, all analyses were performed using TOMTEC-ARENA^®^.

^a^ Refers to the 2004 ISHLT revised classification.

^b^ All measurements were available for these 6 patients.

^c^ Rejection (2R-3R) vs. no significant rejection (0R-1R).

^d^ Mann-Whitney test.

^e^ Unpaired t-test.

Abbreviations: CO, cardiac output; SPAP, systolic pulmonary arterial pressure; PAWP, pulmonary artery wedge pressure; RAP, right atrial pressure; PVR, pulmonary vascular resistance; WU, wood units; CAV, cardiac allograft vasculopathy; LV, left ventricle; LVEF, left ventricular ejection fraction; LVEDV, left ventricular end-diastolic volume; LVESV, left ventricular end-systolic volume; LVSV, left ventricular stroke volume; GLS, global longitudinal strain; SA, semi-automatic; avg, averaged; FA, fully automatic; RV, right ventricle; TAPSE, tricuspid annular peak systolic excursion; RVFWSL, RV free wall strain; RV4CSL, RV global longitudinal strain; LA, left atrium; LASr, LA reservoir strain; LAScd, LA conduit strain; LASct, LA contraction strain; RA, right atrium; RASr, RA reservoir strain; RAScd, RA conduit strain; RASct, RA contraction strain.

#### Association of Echocardiographic Parameters With Outcomes

We analyzed the OR of all echocardiographic parameters for the prevalence of any degree of rejection (serial grading, see *Materials and Methods* under *Outcomes a)*) using logistic regression analysis (Table 3). Only FA RV4CSL was significantly associated with any degree of rejection (OR 1.18, 95% CI 1.02–1.41, P = 0.03; AUC 0.69, P = 0.02) (Supplementary Figure S2). In a group-wise comparison, RV4CSL values demonstrated a stepwise impairment from patients without rejection (0R) to those with 1R, 2R, and 3R (Supplementary Figure S3), although these differences were not statistically significant. All other parameters did not significantly differ, which remained unchanged if only recordings with good quality (level 2) were used for the analyses.

**TABLE 3:** Univariate logistic regression analysis of echocardiographic parameters and any rejection after heart transplantation.

Outcome: Rejection 1R-3R					
Variable	OR	95% CI	P value	AUC	P value
LVEF biplane	0.97	0.87–1.06	0.5	0.58	0.3
LVEDV biplane	1.01	0.99–1.04	0.3	0.64	0.08
LVESV biplane	1.04	0.99–1.10	0.1	0.61	0.2
LVSV biplane	1.01	0.98–1.04	0.6	0.62	0.2
LV Strain (GLS)					
SA-peak avg	1.03	0.88–1.20	0.8	0.52	0.8
FA-peak avg	1.00	0.90–1.11	0.96	0.59	0.3
RV Strain					
SA-RVFWSL	1.07	0.96–1.20	0.3	0.63	0.1
SA-RV4CSL	1.06	0.93–1.23	0.4	0.60	0.2
FA-RVFWSL	1.11	1.01–1.25	0.05	0.71	0.01
FA-RV4CSL	1.18	1.02–1.41	0.03	0.69	0.02
TAPSE	1.08	0.91–1.30	0.4	0.57	0.4
LA Analyses					
SA-LASr	0.99	0.95–1.04	0.8	0.52	0.8
SA-LAScd	0.99	0.93–1.05	0.7	0.54	0.6
SA-LASct	1.03	0.96–1.12	0.4	0.57	0.4
FA-LASr	1.02	0.96–1.09	0.5	0.55	0.5
FA-LAScd	0.96	0.88–1.05	0.4	0.58	0.3
FA-LASct	1.00	0.89–1.13	0.95	0.52	0.8
RA Analyses					
SA-RASr	0.98	0.92–1.03	0.4	0.61	0.2
SA-RAScd	0.98	0.91–1.05	0.6	0.55	0.5
SA-RASct	1.08	1.00–1.19	0.07	0.70	0.01

LV, left ventricle; LVEF, left ventricular ejection fraction; LVEDV, left ventricular end-diastolic volume; LVESV, left ventricular end-systolic volume; LVSV, left ventricular stroke volume; GLS, global longitudinal strain; SA, semi-automatic; avg, average; FA, fully automatic; RV, right ventricle; TAPSE, tricuspid annular peak systolic excursion; RVFWSL, RV free wall strain; RV4CSL, RV global longitudinal strain; LA, left atrium; LASr, LA reservoir strain; LAScd, LA conduit strain; LASct, LA contraction strain; RA, right atrium; RASr, RA reservoir strain; RAScd, RA conduit strain; RASct, RA contraction strain.

**FIGURE 3:**
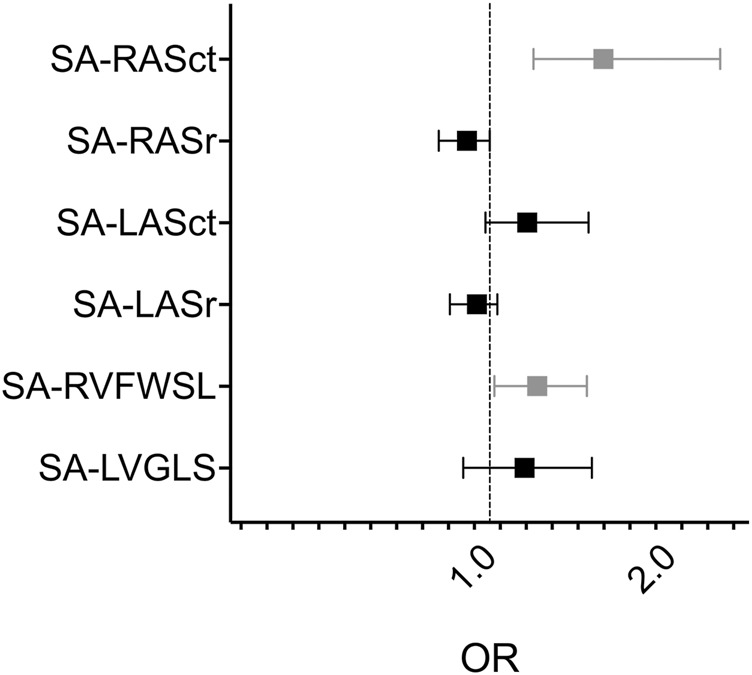
Forest plot of univariate logistic regression analysis of selected semiautomatic strain parameters and rejection 2R - 3R SA, semi-automatic; RAS, right atrial strain; ct, contraction; r, reservoir; LAS, left atrial strain; RVFWSL, right ventricular free wall longitudinal strain; LVGLS, left ventricular global longitudinal strain, OR, odds ratio. Grey datapoints represent significant parameters (SA-RASct: OR 1.55, 95%CI 1.18–2.43, P = 0.01; SA-RVFWSL: OR 1.20, 95%CI 1.02–1.46, P = 0.04).

Using only clinically relevant rejection as the outcome (2R or 3R, Material and *Methods* under *Outcomes b)*), SA-RVFWSL (OR 1.20, 95% CI 1.02–1.46, P = 0.04; AUC 0.79, P = 0.02), SA-RV4CSL (OR 1.27, 95% CI 1.03–1.65, P = 0.04; AUC 0.76, P = 0.04), and RASct (OR 1.55, 95% CI 1.18–2.43, P = 0.01; AUC 0.92, P < 0.001) showed significant associations. All other parameters tested were not significantly different based on the AUC P-value (Table 4; Figure 3).

**TABLE 4:** Univariate logistic regression analysis of echocardiographic parameters and rejection 2R-3R after heart transplantation.

Outcome: Rejection 2R-3R					
Variable	OR	95% CI	P value	AUC	P value
LVEF biplane	0.88	0.76–1.01	0.08	0.72	0.08
LVEDV biplane	1.00	0.97–1.03	0.8	0.56	0.6
LVESV biplane (mL)	1.06	0.98–1.14	0.2	0.67	0.2
LVSV biplane (mL)	0.98	0.92–1.03	0.5	0.57	0.6
LV Strain (GLS)					
SA-peak avg	1.14	0.90–1.48	0.3	0.61	0.4
FA-peak avg	1.05	0.90–1.19	0.5	0.68	0.2
RV Strain					
SA-RVFWSL	1.20	1.02–1.46	0.04	0.79	0.02
SA-RV4CSL	1.27	1.03–1.65	0.04	0.76	0.04
FA-RVFWSL	1.13	0.98–1.32	0.09	0.69	0.1
FA-RV4CSL	1.24	1.01–1.56	0.04	0.72	0.09
TAPSE	1.17	0.89–1.58	0.3	0.62	0.4
LA Analyses					
SA-LASr	0.95	0.86–1.03	0.3	0.65	0.2
SA-LAScd	1.01	0.92–1.14	0.8	0.51	0.96
SA-LASct	1.16	0.98–1.46	0.2	0.70	0.1
FA-LASr	0.97	0.87–1.06	0.5	0.57	0.6
FA-LAScd	1.00	0.88–1.15	0.99	0.52	0.9
FA-LASct	1.13	0.93–1.46	0.3	0.64	0.3
RA Analyses					
SA-RASr	0.92	0.82–1.00	0.07	0.74	0.06
SA-RAScd	0.98	0.89–1.10	0.8	0.50	1.0
SA-RASct	1.55	1.18–2.43	0.01	0.92	<0.001

LV, left ventricle; LVEF, left ventricular ejection fraction; LVEDV, left ventricular enddiastolic volume; LVESV, left ventricular end-systolic volume; LVSV, left ventricular stroke volume; GLS, global longitudinal strain; SA, semi-automatic; avg, average; FA, fully automatic; RV, right ventricle; TAPSE, tricuspid annular peak systolic excursion; RVFWSL, RV free wall strain; RV4CSL, RV global longitudinal strain; LA, left atrium; LASr, LA reservoir strain; LAScd, LA conduit strain; LASct, LA contraction strain; RA, right atrium; RASr, RA reservoir strain; RAScd, RA conduit strain; RASct, RA contraction strain.

**FIGURE 4:**
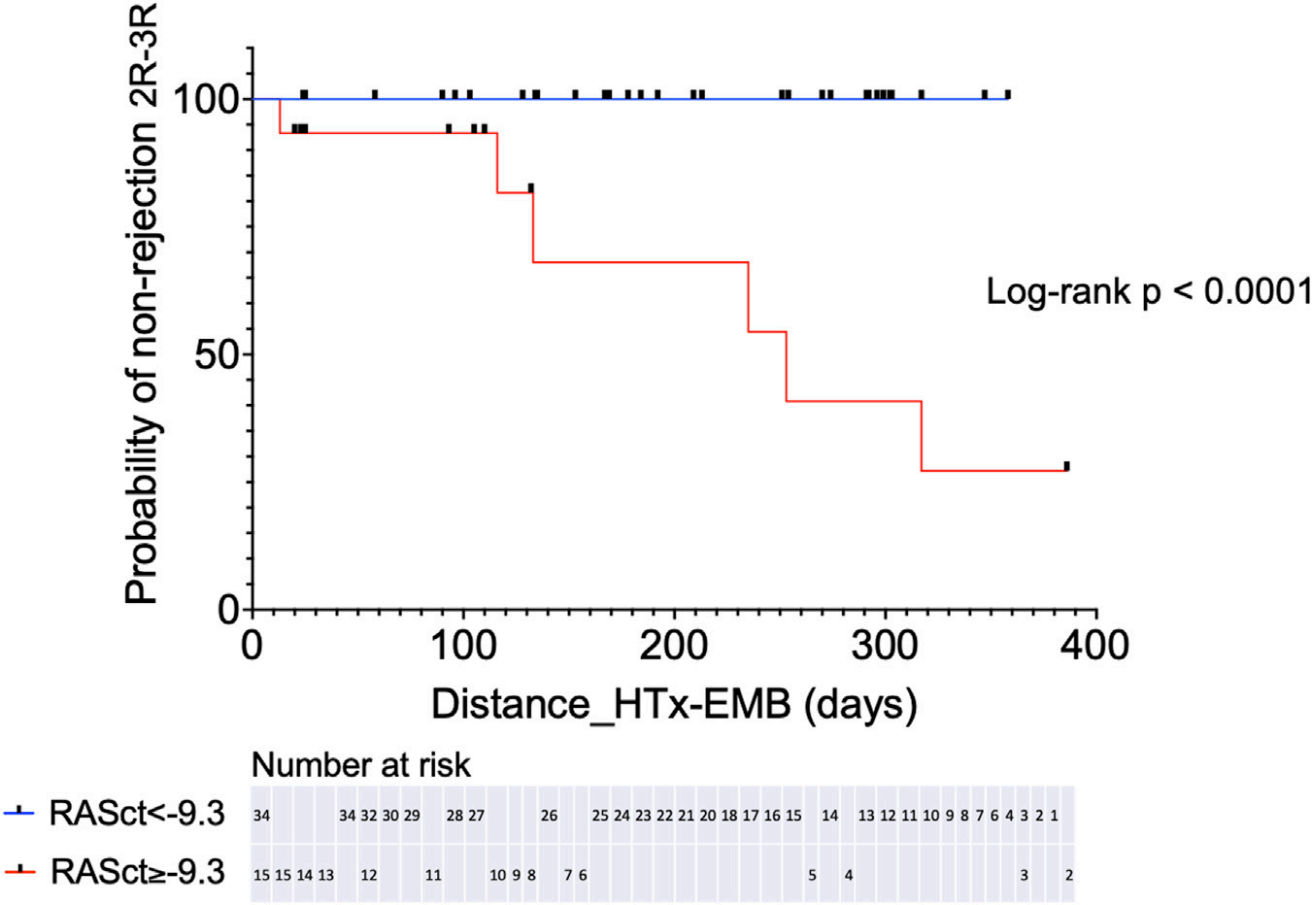
Kaplan-Meier analysis for probability of non-rejection 2R-3R in two groups separated by a chosen cut-off value for RASct (right atrial contraction strain). HTx, heart transplantation; EMB, endomyocardial biopsy.

We further assessed the prognostic significance of RASct, which emerged as the parameter with the most robust association with rejection. A cut-off value of <−9.3% was chosen because of its sensitivity of 100% and a negative predictive value of 100% for rejection 2R or 3R (specificity 79%, positive predictive value 40%). Kaplan-Meier analysis showed a very pronounced difference in the probability of freedom of significant rejection (2R or 3R) within 1 year after HTX between patients with an RASct <−9.3% (indicating better RA contractility) vs. those patients with an RASct ≥−9.3% (log-rank HR 0.000, P < 0.0001) (Figure 4).

### Analysis 2

#### Association of Intraindividual Echocardiographic Changes and Rejection

**FIGURE 5:**
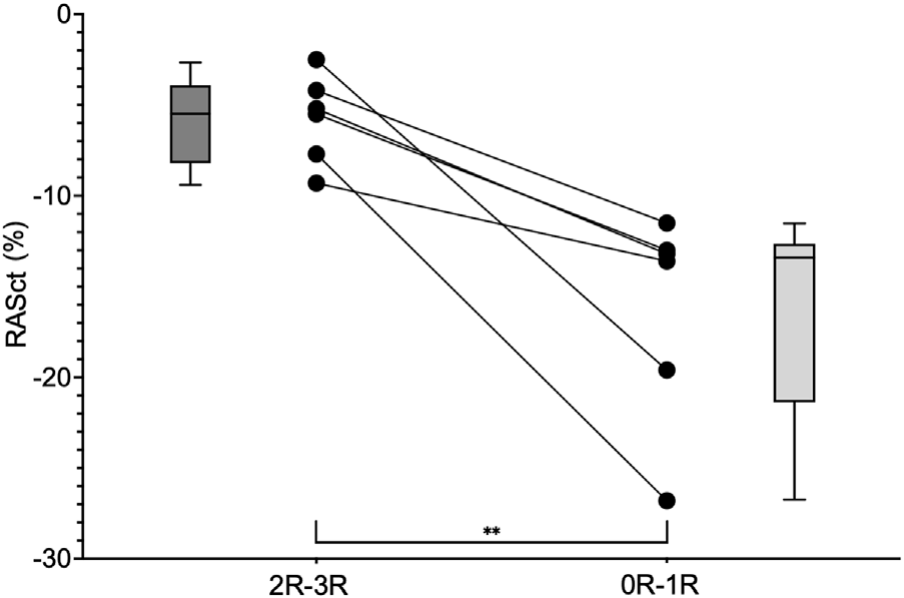
Groupwise comparison of RASct values (minimum, maximum and median) at the time of rejection (2R-3R) and without relevant rejection (0R-1R) in six individuals. RASct, right atrial contraction strain. ** = P = 0.008.

**FIGURE 6:**
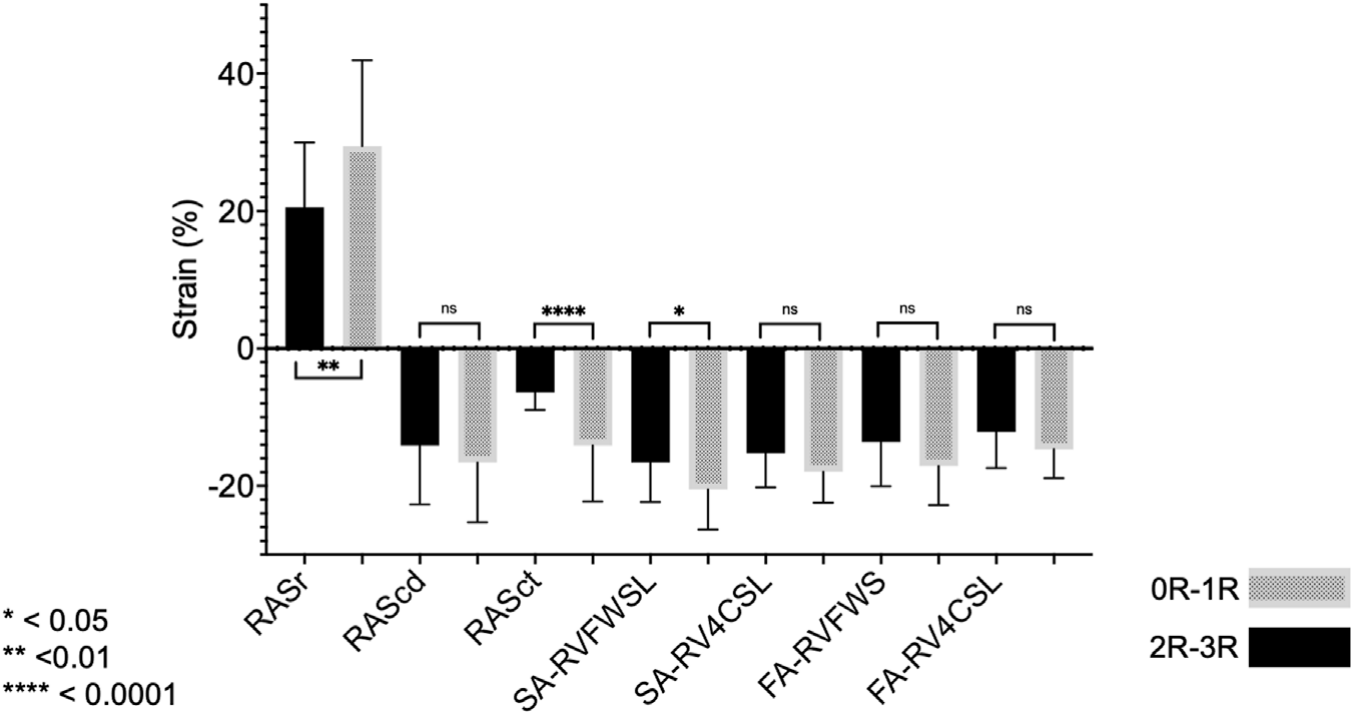
Analysis 2 (extended samples): groupwise comparisons of different strain parameters corresponding to EMB samples with 0R-1R (n = 48) vs. 2R-3R (n = 14). Note that reservoir strain has a positive value, and a higher value indicates better reservoir function. The opposite is true for the other strain parameters. For abbreviations, please refer to the above figures.

Twenty-seven patients met our inclusion criteria of having paired results of one EMB without rejection (0R) and one following EMB with rejection (1R-3R). Overall, the analysis of this population did not reveal echocardiographic differences between these two EMB statuses. Therefore, we further restricted our analysis to those six individuals with clinically significant rejection (2R-3R) and compared their findings to when they were without significant rejection (0R-1R) (Figure 5). A significant change in RASct was observed between the situation of rejection and that of no rejection in these individuals (P = 0.008). In a further modified analysis (“extended samples”), instead of pairs of EMB, we analyzed pooled SE results of 2R-3R vs. 0R-1R in our HTx population (Figure 6). Accordingly, we chose each EMB with 2R-3R from the patients of the original Analysis 2 (the maximum was six relevant rejections in one single patient) and one additional patient with 3R, all in all 14 EMB with 2R-3R. These were compared with all EMB 0R-1R from the original Analysis 2 (27 0R + 21 1R). Groupwise comparisons showed significant differences in RASr (P = 0.007), RASct (P < 0.0001), and SA-RVFWS (P = 0.03).

### Analysis 3

The validation cohort comprised 23 patients (out of 25 screened, 2 did not have echocardiograms of sufficient quality available). Three of 23 had rejection requiring therapy, and their corresponding RASct values were all ≥−9.3% (median −5.1%). EMB of those 3 patients at another point in time without the need for therapy (0R-1R) were corresponding to RASct values <−9.3% (median −13.4%). Of the other 20 patients with EMB 0R-1R, 19 had RASct values <−9.3% and one patient had −8.0% (median of the whole group −13.75%). In summary, 95.7% of all 23 EMB 0R-1R in this cohort corresponded to RASct values <−9.3%.

## Discussion

We present a comprehensive analysis of the standard and strain echocardiographic parameters of all cardiac chambers to evaluate subclinical ACR after HTx. The relevant findings of our study are as follows: (i) conventional parameters such as LVEF, LV stroke volume, and TAPSE did not show any association with ACR in our cohort; (ii) the association between impaired RV strain and ACR was confirmed; (iii) we identified a robust association between RASct and clinically relevant ACR (2R-3R) that was not present in the LA strain; (iv) the use of a recently developed dedicated SE software may partly overcome previous limitations due to the variability of measurements caused by the necessity for manual corrections of endocardial tracking.

Echocardiography has been used for routine surveillance after HTx for decades, and the use of Doppler tissue imaging for this purpose has been discussed since the late 1990s [24, 25]. Positive results for SE of the LV and RV for ACR evaluation have been reported [11–16], but there are also ambivalent results [26–29]. Furthermore, the impact of inter-vendor variability and the lack of standardization of the parameters to be measured and dedicated software packages for obtaining such measurements are important issues [17, 18, 30]. In summary, the diagnostic value of echocardiographic myocardial deformation imaging for detection of ACR after HTx is quite variable so far [31], and our negative results for SE of the LV are most likely to be seen against this background.

In comparison with reference ranges for strain in a healthy population, HTx patients show significantly lower ventricular and left atrial strain values early after transplantation in the absence of relevant rejection [17, 22, 32–36]. Reference ranges for RA strain in HTx patients have not been published; in our study, SE measurements of all four chambers were lower than those published in healthy controls. There was a high rate of concordance between serial measurements using up-to-date SE analysis software in our cohort (quality level 2 in 87% of measurements), which makes the current software version significantly different from the previous version in our experience. FA measurements in general showed markedly worse ventricular strain results than measurements made in the SA mode; for the LA, this was true only for reservoir and contraction strain, whereas conduit strain was identical. The RA strain was only available in the SA mode. Overall, there was a certain, albeit low, rate of dropouts due to insufficient FA endocardial demarcation, and SA measurements seemed more realistic to us. Furthermore, the SA mode produced more significant results in our study. Accordingly, we prefer the SA mode for the analysis of strain measurements.

A recent study investigated the role of LA longitudinal strain (LALS) in the non-invasive diagnosis of ACR episodes in HTx recipients. LALS variables principally discriminated between studies without rejection and those with any grade of ACR, but reproducibility between comparable LALS parameters was poor, and inter-vendor variability was significant [17]. To the best of our knowledge, our study is the first investigation of SE of the RA for ACR screening. In contrast, there is a growing body of literature concerning RA SE in heart failure and pulmonary hypertension [37, 38]. We found an impaired RASct to be strongly associated with clinically relevant ACR, and based on the main target to avoid futile EMB, we chose a cut-off value enabling the identification of patients not in need of EMB. Patients with RASct values below this value had a 100% probability of freedom from relevant ACR. This was achieved at the cost of specificity, so that the positive predictive value for ACR was low, which is very similar to the findings of several studies evaluating ventricular SE and ACR [16]. In uncertain cases, EMB will be undoubtedly still essential. However, patients with preserved RASct and no clinical suspicion of ACR could be spared from unnecessary EMB.

However, why should RA contractility, in particular, be useful for ACR evaluation? This thin-walled structure can be seen as “the weakest link in the chain,” as it is exposed to only low pressures in the healthy circulation and may have heightened vulnerability for different hazards. The LA is also normally exposed to low pressures, but diastolic LV dysfunction occurs early after HTx [39] and may increase the LA load and thus its muscularization. However, in our study, there is one obvious obstacle that may lead to a situation in which LA strain is prone to be incorrect. While all our patients received a bicaval anastomosis at HTx with complete resection of the recipient RA, the donor LA was anastomosed using the usual technique to a remnant of the recipient LA. Accordingly, there was an enlargement of the long-axis dimension of the LA with a ridge at the site of anastomosis, leading to difficult or even impossible measurement of correct SE parameters [27, 40]. In one study, LA function was generally worse in HTx patients than in controls [41]. As a consequence, measurement of LA strain may not be of particular use in HTx recipients.

### Limitations

Given the retrospective, non-randomized study design and the limited sample size, our findings should be interpreted with caution. In the confirmatory Analysis 2, we attempted to expand the findings of Analysis 1 to an extended number of EMB so that our results could be reproduced and adapted to the intraindividual course. Image quality of echocardiograms was judged to be insufficient in 16% of patients initially screened, which is a relatively high rate and limits applicability in clinical practice. Furthermore, an elaborate procedure was necessary to achieve reproducible results. Even though the semi-automatic approach enables to get timely results of single measurements, the whole process is more time consuming than routine measurements. However, this effort could be worthwhile to avoid unnecessary EMB. The SE of the RA with the software used is formally off-label; however, it is based on the same principles as LA strain, its application is straightforward with high reproducibility, and other groups have taken a similar approach.

Published reference ranges for RA contractile strain have shown wide confidence intervals including values similar to those we found in patients with significant rejection [22, 42]. However, within the overall range, these values were at the extreme edge of the spectrum. Therefore, an approach using better RA contractile strain as a clue for absence of significant rejection would be compatible with this.

Antibody-mediated rejection was not the subject of this study but was not present in any of our patients. Our findings should be regarded as hypothesis generating and applied to a greater number of HTx patients in future studies.

## Conclusion

In the times of gene expression profiling and DNA analysis for rejection monitoring, the utility of echocardiography may be underestimated, and SE may have been underused for reasons of practicability and lack of reliability. RA strain analysis using recent technical developments may be a promising tool for reducing the likelihood of subclinical ACR after HTx and for avoiding futile EMB. In accordance with the ISHLTguideline statement “an echo-supported minimization of biopsy surveillance appears the optimal approach” [1], analysis of RA contraction strain could be a step towards further EMB minimization. However, this should be investigated in future, prospective studies.

## Data Availability

The raw data supporting the conclusions of this article will be made available by the authors, without undue reservation.

## Abbreviations

ACR, acute cellular rejection Serial grading of ACR; 0R, no rejection; 1R, mild rejection; 2R, moderate rejection; 3R, severe rejection; EMB, endomyocardial biopsy; FA, fully automatic; HTx, heart transplantation; ISHLT, International Society for Heart and Lung Transplantation; LA, left atrial; LV, left ventricular; LVEF, left ventricular ejection fraction; RA, right atrial; RV, right ventricular; RV4CSL, right ventricular global 4-chamber contour strain longitudinal; RVFWSL, right ventricular free wall strain longitudinal; SA, semi-automatic; Sc, conduit strain; Sct, contraction strain; SE, speckle tracking-derived strain echocardiography; Sr, reservoir strain; TAPSE, tricuspid annular plane systolic excursion.
