# Supplementary material for: Right Atrial Contraction Strain Is Associated With Clinically Significant Cellular Rejection in Patients After Heart Transplantation
Source: Transpl Int. 2025 Oct 16;38:14174. doi: 10.3389/ti.2025.14174 (PMC12571671; doi:10.3389/ti.2025.14174)
Supplement: Supplementary file 1 [file DataSheet1.pdf]

SUPPLEMETARY MATERIAL

Table of contents:

Title	Content	Page
Supplementary Figure S1	CONSORT Flow Diagram	2
Supplementary Figure S2	Forest plot of different RV strain measurements and any rejection	3
Supplementary Figure S3	Groupwise comparisons of RV4CSL in different rejection grades	4
Capsule Sentence Summary		

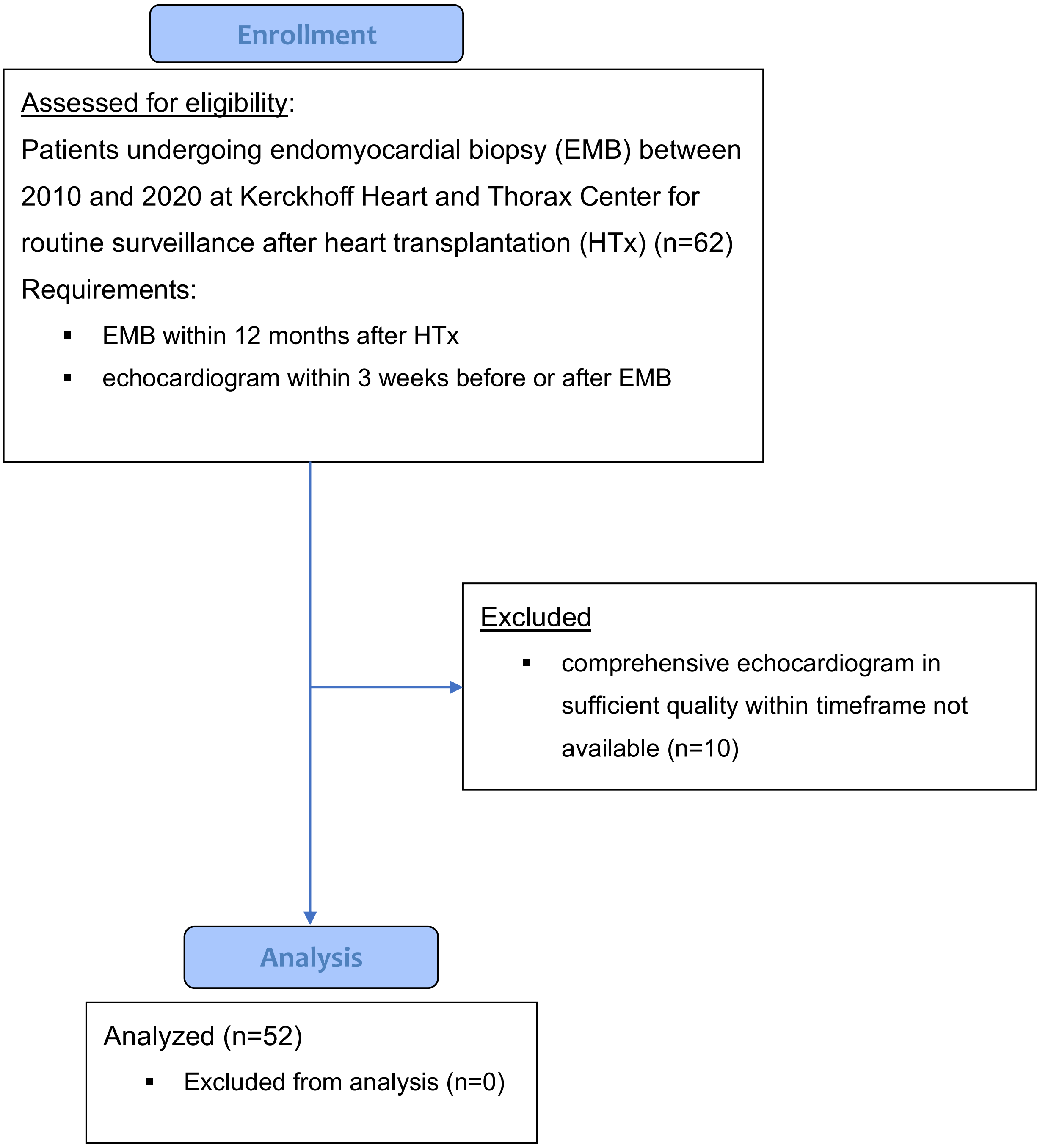

Supplementary Figure S1: CONSORT Flow Diagram

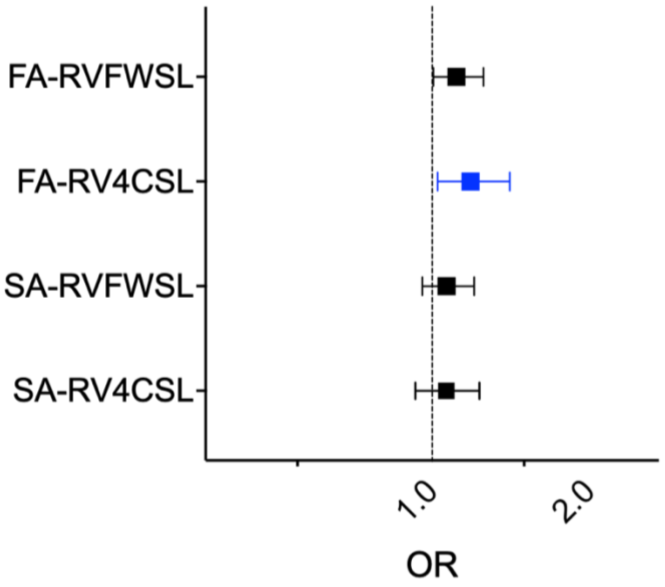

Supplementary Figure S2: Forest plot of different RV strain measurements and any rejection (logistic regression analysis).

FA-RVFWS = fully automatic right ventricular free wall longitudinal strain; FA-RV4CSL = fully automatic right ventricular global 4-chamber contour longitudinal strain; SA= semi-automatic, OR = odds ratio. Blue datapoint represents significant parameters (FA-RVFWSL: OR 1.18, 95%CI 1.02-1.41, P = 0.03).

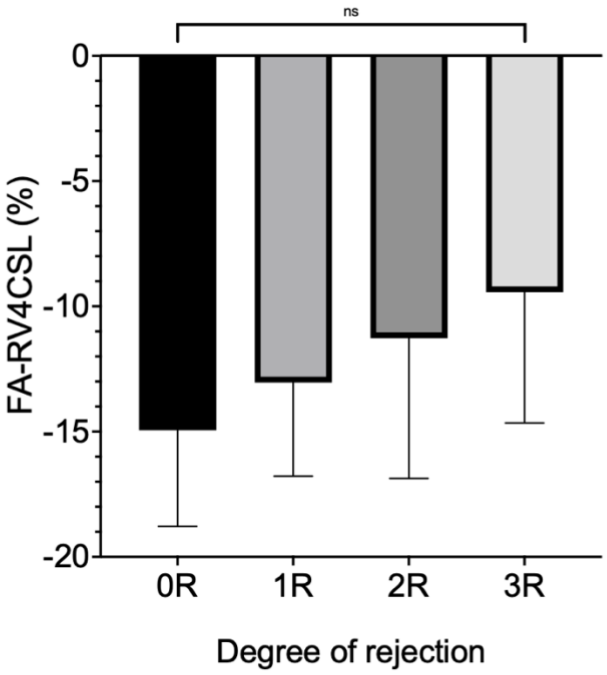

Supplementary Figure S3: Groupwise comparisons of RV4CSL in different rejection grades.

FA-RV4CSL = fully automatic right ventricular global 4-chamber contour longitudinal strain; 0R = rejection ISHLT 0R, etc. ns = not significant.

Capsule Sentence Summary

Associations between strain echocardiography and results of endomyocardial biopsy were assessed in patients after heart transplantation. Right atrial contraction strain using a certain threshold value was able to rule out clinically significant myocardial rejection almost 100 percent.
